# Trends in radiotherapy use and implementation challenges among patients with cervical cancer: a multicenter study in Osaka, Japan

**DOI:** 10.1093/jrr/rraf066

**Published:** 2025-11-10

**Authors:** Toshiki Ikawa, Toshitaka Morishima, Kayo Nakata, Kenji Kishimoto, Setsuo Tamenaga, Naoyuki Kanayama, Masahiro Morimoto, Koji Konishi, Isao Miyashiro

**Affiliations:** Cancer Control Center, Osaka International Cancer Institute, 3-1-69 Otemae, Chuo-ku, Osaka 541-8567, Japan; Department of Radiation Oncology, Osaka International Cancer Institute, 3-1-69 Otemae, Chuo-ku, Osaka 541-8567, Japan; Cancer Control Center, Osaka International Cancer Institute, 3-1-69 Otemae, Chuo-ku, Osaka 541-8567, Japan; Cancer Control Center, Osaka International Cancer Institute, 3-1-69 Otemae, Chuo-ku, Osaka 541-8567, Japan; Cancer Control Center, Osaka International Cancer Institute, 3-1-69 Otemae, Chuo-ku, Osaka 541-8567, Japan; Department of Radiation Oncology, Osaka International Cancer Institute, 3-1-69 Otemae, Chuo-ku, Osaka 541-8567, Japan; Department of Radiation Oncology, Osaka International Cancer Institute, 3-1-69 Otemae, Chuo-ku, Osaka 541-8567, Japan; Department of Radiation Oncology, Osaka International Cancer Institute, 3-1-69 Otemae, Chuo-ku, Osaka 541-8567, Japan; Department of Radiation Oncology, Osaka International Cancer Institute, 3-1-69 Otemae, Chuo-ku, Osaka 541-8567, Japan; Cancer Control Center, Osaka International Cancer Institute, 3-1-69 Otemae, Chuo-ku, Osaka 541-8567, Japan

**Keywords:** cervical neoplasms, radiotherapy, brachytherapy, healthcare demand, trends

## Abstract

Since 2018, the staging system and guidelines for cervical cancer have been revised in Japan. Here, we analyzed trends in radiotherapy use among patients with cervical cancer in Osaka Prefecture, Japan. We obtained records from hospital-based cancer registries (2016–23) linked to Diagnosis Procedure Combination data (2019–23), from 67 nationally or prefecturally designated cancer care hospitals. Eligible patients had epithelial or neuroendocrine cervical cancer, excluding those with clinical stage 0 or unknown clinical stage with pathological stage 0. Between 2016 and 2023, the number of patients per year remained stable (717–787); the number of stage IB–IIA (FIGO 2018) cases decreased, whereas that of stages IIB or IIIC (T1–2) cases increased. The number of patients receiving radiotherapy as initial treatment increased from 229 in 2016 to 294 in 2023; this was accompanied by a decline in surgical treatment. The proportion of patients undergoing radiotherapy increased from 11.9% to 17.2% for stage IB–IIA, from 55.6% to 71.7% for stage IIB and from 38.0% to 69.5% for stage IIIC (T1–2). Among 11 institutions providing brachytherapy, the number of radiotherapy cases increased at three, whereas it remained stable or declined at the other eight. These findings indicate a growing trend in radiotherapy use for cervical cancer in Osaka Prefecture; however, the increase varied by institution. To sustain cervical cancer radiotherapy services, further studies may be needed to assess the adequacy of brachytherapy staffing, explore the financial feasibility of brachytherapy equipment, and examine the potential implications of brachytherapy centralization.

## INTRODUCTION

Cervical cancer is one of the most prevalent malignancies among women in Japan. Despite being preventable through vaccination against human papillomavirus and screening [1], these measures remain insufficient in Japan [2, 3]. The estimated incidence was 7868 cases in 2000, which increased to 10 737 cases in 2010, with 10 353 cases reported in 2020 [4, 5].

The standard treatment for nonmetastatic cervical cancer includes radical surgery with adjuvant therapy based on the pathological risk or definitive radiotherapy with or without chemotherapy [6]. Since 2018, major updates to the staging system and Japanese guidelines have been made, affecting treatment choices. That year, the International Federation of Gynecology and Obstetrics (FIGO) revised the cervical cancer staging system [7], which was subsequently adopted by the Japan Society of Gynecologic Oncology in its 2022 guidelines [6]. These revisions have enabled the integration of imaging findings into stage classification, highlighting the key imaging factors that guide the selection of radiotherapy over surgery, such as parametrial invasion (stage IIB) and lymph node metastasis (stage IIIC1, pelvic; stage IIIC2, para-aortic). The National Comprehensive Cancer Network (NCCN) guidelines recommend radiotherapeutic management for both stages IIB and IIIC1–2 [8].

Furthermore, the recommendations for stages IIB and III cervical cancer in the Japanese guidelines have also been updated. The 2017 guidelines, based on the FIGO 2009 staging system, recommended surgical management for stage IA, surgical or radiotherapeutic management for stage IB–IIB and radiotherapeutic management for stage III–IVA [9]. The 2022 guidelines, based on the FIGO 2018 staging system, revised the stage IIB recommendations for radiotherapeutic management [6]. However, unlike the NCCN guidelines, the Japanese guidelines propose both surgical and radiotherapeutic management of stage IIIC1 (T1–2) cervical cancer, which is characterized by pelvic lymph node metastasis with T1–2 tumors.

Changes in staging systems and guidelines have likely expanded the role of radiotherapy in cervical cancer treatment in Japan, thereby increasing the clinical need for radiotherapy. An Annual Report published by the Committee on Gynecologic Oncology of the Japan Society of Obstetrics and Gynecology showed that radiotherapeutic management accounted for 35.4% of all cervical cancer treatments in 2017, which increased to 38.3% in 2021 [10, 11]. However, the influence of these updates on radiotherapy selection remains unclear. Assessing this effect requires an analysis of the trends in the proportion of patients receiving radiotherapy at each stage.

Radiotherapy for cervical cancer typically consists of external beam therapy, followed by brachytherapy [12]. However, brachytherapy is only available at a limited number of institutions in Japan (177 institutions, according to a 2022 survey [13]). Facilities without brachytherapy equipment either refer patients elsewhere for treatment or provide external beam radiotherapy followed by a referral for brachytherapy. As the clinical need for cervical cancer radiotherapy increases, brachytherapy centers may face a capacity strain. A 2022 survey in Japan [13] found that 43% of facilities were operating at maximum capacity, and 4% had already exceeded their limits. Evaluating regional radiotherapy requirements is vital to the continued treatment of cervical cancer.

This study evaluated the trends in radiotherapy use among patients with cervical cancer using hospital-based cancer registry records from Osaka Prefecture, which has the third largest population in Japan (8.8 million in 2023). Furthermore, we discussed challenges related to the implementation of cervical cancer radiotherapy in this region.

## MATERIALS AND METHODS

### Study design and population

This observational study was conducted as part of the cancer registry–based study on cancer care in Osaka (CanReCO), a project led by the Council for the Coordination of Designated Cancer Care Hospitals in Osaka. CanReCO included hospital-based cancer registry records for patients diagnosed from 2016 to 2023, with Diagnosis Procedure Combination (DPC) records linked to the registry for cases from 2019 to 2023. These data were collected from all nationally or prefecturally designated cancer care hospitals in Osaka, which cover 80–90% of cancer surgery or endoscopic resection cases in the region [14]. The hospital-based cancer registry includes patient-level data on sex, age at diagnosis, year of diagnosis, tumor site and histology codes based on the International Classification of Diseases for Oncology third edition (ICD-O-3), Union for International Cancer Control (UICC) tumor-node-metastasis (TNM) classification and initial treatments, including surgery, radiotherapy and systemic therapy. DPC records contain claims data on inpatient and outpatient services, including radiotherapy dates and techniques.

We analyzed the anonymized data from 67 designated cancer care hospitals, excluding those newly designated or delisted between 2016 and 2023. Patients were included if they had an ICD-O-3 site code of C53 and a histological code classified as epithelial or neuroendocrine carcinoma. Neuroendocrine carcinoma was included in the analysis because patients with this diagnosis are generally treated with combined local and systemic therapy, as is the case with epithelial tumors [8]. The histological codes included those for epithelial tumors of cervical origin, based on the Rare Cancer Classification Table from the Surveillance, Epidemiology, and End Results Program [15], along with additional codes 8077, 8013, 8041, 8045 and 8246. We excluded patients with clinical stage 0 and those with unknown clinical stage but pathological stage 0. The clinical TNM classification recorded in the hospital-based cancer registries using the UICC staging system was converted to the FIGO 2018 staging system. In hospital-based cancer registries, para-aortic lymph node metastases were recorded as distant metastases in 2016–17 and as regional lymph node metastases from 2018 onward. The registries lack information to identify para-aortic lymph node metastases; therefore, cT3N0M0 and cT1–3N1M0 cases were classified as stage III and cT1–4 N0–1M1 was classified as stage IVB throughout the study period. Stage III was subclassified according to the clinical T category: cT1–2N1M0 was classified as stage IIIC (T1–2) and cT3N0–1 M0 as stage III (T3).

This study was approved by the ethics committee of Osaka International Cancer Institute (approval number 24196). The requirement for patient consent for the CanReCO project was waived because the data were collected for health policy planning and research purposes using an opt-out approach at the participating hospitals.

### Statistical analyses

Descriptive statistics were used to analyze trends in treatment use and stage distribution by year of diagnosis among patients with cervical cancer. First, we reviewed the annual number of patients according to initial treatment and stage at diagnosis. Initial treatment was classified as one of the following: surgery (with or without radiotherapy and/or systemic therapy), radiotherapy (with or without systemic therapy), systemic therapy alone or other (no treatment or unknown treatment details). Second, we assessed the annual proportion of patients receiving radiotherapy among those undergoing surgery or radiotherapy for stages IB–IIA, IIB and IIIC (T1–2) cervical cancer.

Third, we assessed facility-level changes in the number of patients undergoing radiotherapy (2016–23) and brachytherapy (2019–23, when DPC data were available). The analysis was limited to 11 institutions that reported providing brachytherapy to patients with cervical cancer in a 2024 survey conducted by the Japanese Group of Brachytherapy/Japanese Society for Radiation Oncology [16]. Because hospital-based cancer registries lack unique patient identifiers across institutions, patients who began radiotherapy at one facility and were referred to another for additional radiotherapy procedures (e.g. brachytherapy) were recorded as radiotherapy cases at both institutions. Brachytherapy was classified as intracavitary or interstitial therapy. Patients who received both types during multiple sessions were categorized as receiving interstitial therapy. Figures were generated using the R software (version 4.2.3) (R Foundation for Statistical Computing, Vienna, Austria).

## RESULTS

From 2016 to 2023, a total of 5995 patients were included in the study. Among them, 3331 underwent surgery and 2057 received radiotherapy as initial treatment. Patient characteristics are summarized in Table 1. Patients in the radiotherapy group were older (median age, 65 vs 47 years) and had stage IIB or higher disease more frequently compared with those in the surgery group.

**Table 1:** Patient characteristics

Characteristic	Total, *n* = 5995	Surgery, *n* = 3331	Radiotherapy, *n* = 2057	Systemic therapy, *n* = 302	Other, *n* = 305
Age at diagnosis, years	54 (43, 69)	47 (39, 59)	65 (51, 76)	59 (50, 71)	80 (61, 87)
Stage (FIGO 2018)					
IA	761 (12.7%)	724 (21.7%)	2 (0.1%)	0 (0.0%)	35 (11.5%)
IB–IIA	2198 (36.7%)	1861 (55.9%)	296 (14.4%)	7 (2.3%)	34 (11.1%)
IIB	697 (11.6%)	254 (7.6%)	414 (20.1%)	8 (2.6%)	21 (6.9%)
IIIC (T1–2)	737 (12.3%)	275 (8.3%)	436 (21.2%)	18 (6.0%)	8 (2.6%)
III (T3) or IVA	798 (13.3%)	36 (1.1%)	671 (32.6%)	25 (8.3%)	66 (21.6%)
IVB	615 (10.3%)	33 (1.0%)	232 (11.3%)	239 (79.1%)	111 (36.4%)
Unknown	189 (3.2%)	148 (4.4%)	6 (0.3%)	5 (1.7%)	30 (9.8%)
Histology					
Epithelial carcinoma	5910 (98.6%)	3284 (98.6%)	2039 (99.1%)	289 (95.7%)	298 (97.7%)
Neuroendocrine carcinoma	85 (1.4%)	47 (1.4%)	18 (0.9%)	13 (4.3%)	7 (2.3%)

Data are presented as the median (interquartile range) or *n* (%). Patients were stratified into the surgery (surgery ± radiotherapy ± systemic therapy), radiotherapy (radiotherapy ± systemic therapy), systemic therapy alone and other (no treatment or unknown treatment details) groups. FIGO = International Federation of Gynecology and Obstetrics.

**Fig. 1:**
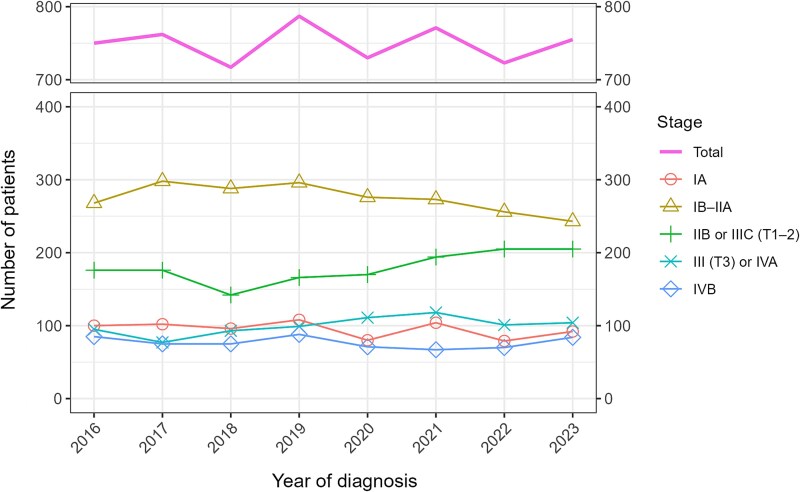
Trends in the number of patients with cervical cancer by stage at 67 designated cancer care hospitals in Osaka Prefecture. The total includes patients with unknown stage or TNM classification.

**Fig. 2:**
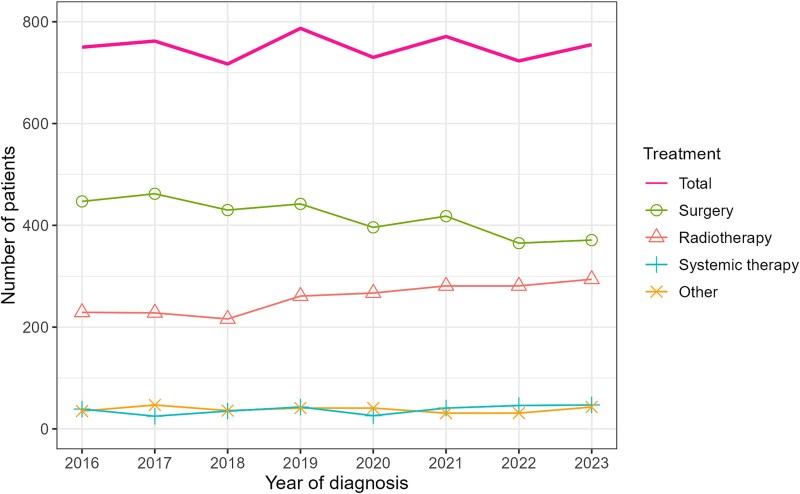
Trends in the number of patients with cervical cancer by initial treatment. Patients were stratified into the surgery (surgery ± radiotherapy ± systemic therapy), radiotherapy (radiotherapy ± systemic therapy), systemic therapy alone and other (no treatment or unknown treatment details) groups.

**Fig. 3:**
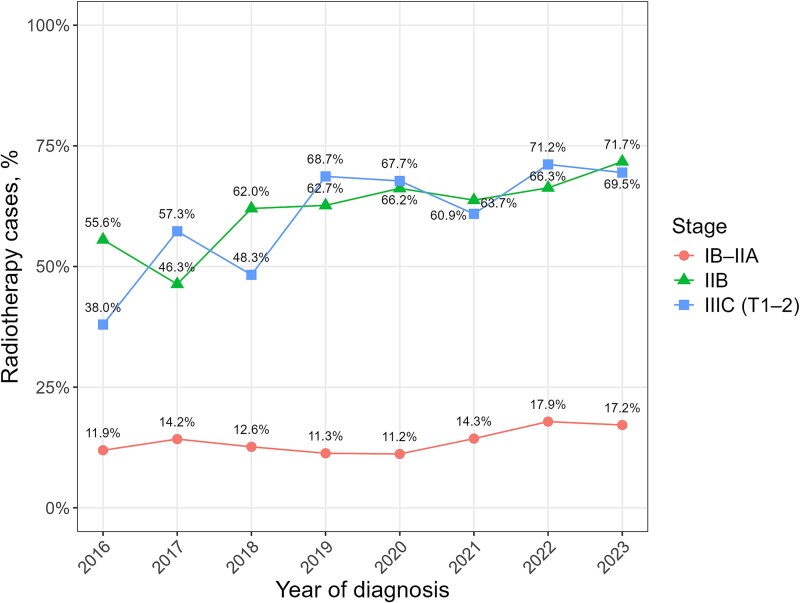
Trends in the proportion of patients receiving radiotherapy among those undergoing surgery or radiotherapy for stages IB–IIA, IIB and IIIC (T1–2) cervical cancer.

The number of patients with cervical cancer (per year) remained between 717 and 787, with no clear trend observed (Fig. 1). From around 2020 onward, the number of stage IB–IIA cases decreased, whereas that of stage IIB or IIIC (T1–2) cases increased (Fig. 1). The number of patients who received radiotherapy as initial treatment increased from 229 in 2016 to 261 in 2019 and to 294 in 2023, whereas the number of surgical cases declined (Fig. 2). Figure 3 shows the trends in the proportions of patients receiving radiotherapy among those undergoing surgery or radiotherapy for stages IB–IIA, IIB or IIIC (T1–2) cervical cancer. From 2016 to 2023, the proportion increased from 55.6% to 71.7% for stage IIB and from 38.0% to 69.5% for stage IIIC (T1–2). In stage IB–IIA, the proportion increased modestly from 2021 onward, from 11.2% in 2020 to 17.2% in 2023.

**Fig. 4:**
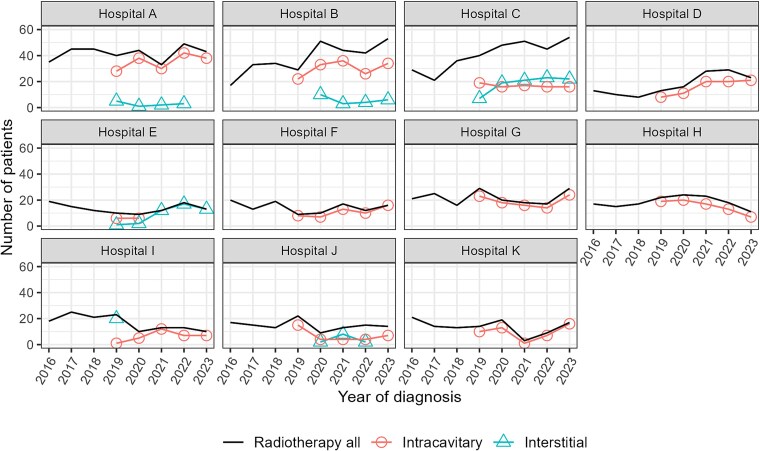
Trends in the numbers of patients with stage I–IV cervical cancer who received radiotherapy as initial treatment and those who underwent brachytherapy (intracavitary or interstitial) at 11 respective institutions. Absence of the triangle symbol indicates zero interstitial brachytherapy cases. Patients who began radiotherapy at one facility and were referred to another for additional radiotherapy procedures (e.g. brachytherapy) were counted as radiotherapy cases at both institutions.

Figure 4 shows the trends in the number of patients who received radiotherapy as initial treatment and those who underwent brachytherapy at the 11 institutions providing brachytherapy. Three institutions (A–C) had the highest treatment volume (~40–50 radiotherapy cases in 2023), accounting for 51% of the brachytherapy cases performed across the 11 facilities in 2023. A notable increase in radiotherapy cases was observed at three institutions (B–D) from around 2019 onward. Compared with the average number of patients from 2016 to 2018, the average number from 2019 to 2023 was 1.6–2.2 times higher. In contrast, in the remaining eight institutions (A and E–K), the number of radiotherapy cases either remained stable or declined. Interstitial brachytherapy was primarily performed at two centers (C and E), accounting for 85% of such cases across 11 institutions in 2023.

## DISCUSSION

This study analyzed the trends in radiotherapy use among patients with cervical cancer at designated cancer care hospitals in Osaka Prefecture, Japan. The number of patients with cervical cancer remained stable from 2016 to 2023. However, the number of patients receiving radiotherapy increased, accompanied by a decline in that of patients who underwent surgical treatment. During this period, the proportion of patients receiving radiotherapy increased compared with that of patients undergoing surgery in the stage IB–IIA, IIB and IIIC (T1–2) groups. The proportion of patients receiving radiotherapy has been reported previously [10, 11]. However, the novelty of this study lies in analyzing the trends in radiotherapy use, specifically within stages IB–IIA, IIB and IIIC (T1–2), for which both surgery and radiotherapy are potential treatment options in Japan.

The use of radiotherapy over surgery for stages IIB and IIIC (T1–2) has been increasing, even before the Japanese guideline revision in 2022. Additionally, since 2021, the proportion of patients receiving radiotherapy has modestly increased even in stage IB–IIA. This trend may reflect the growing recognition of the role of radiotherapy in these stages, which is supported by the reported favorable outcomes of radiotherapeutic management [17–20]. The revision of the 2022 Japanese guidelines may lead to a further increase in the proportion of patients receiving radiotherapy, highlighting the need for continued monitoring.

The number of stage IB–IIA cases decreased, while that of stage IIB and IIIC (T1–2) cases increased. This shift was likely related to the recent revision of the FIGO staging system. In Japan, hospital-based cancer registries follow the UICC TNM classification system, which incorporates imaging findings, whereas clinical guidelines follow the FIGO staging system. The FIGO revision emphasized the incorporation of imaging findings into staging [7], which may have influenced how the registries recorded the tumor stage. Consequently, some patients who would previously have been classified as having stage IB–IIA may now be assigned to higher stages. Additionally, the coronavirus disease 2019 (COVID-19) pandemic, which strained healthcare systems worldwide, may have contributed to changes in stage distribution. Since the onset of the COVID-19 pandemic, the proportion of cervical cancer cases detected through screening has declined, possibly leading to a decrease in early-stage cases and an increase in advanced-stage cases [21].

A facility-level analysis showed that the degree of increase in the number of patients receiving radiotherapy for cervical cancer varied across institutions. Among the 11 institutions providing brachytherapy, 3 showed an increase in radiotherapy patient numbers, whereas the numbers remained stable or declined in the other institutions. One possible reason is that radiotherapy for cervical cancer, particularly brachytherapy, requires a high level of expertise, which leads to increased patient referrals to more experienced institutions. Notably, interstitial brachytherapy was predominantly performed at two facilities. Centralizing treatment in selected hospitals may improve patient outcomes and resource efficiency. However, excessive centralization can lead to regional disparities, limiting access to appropriate care and potentially reducing the availability of brachytherapy.

Brachytherapy is essential in cervical cancer radiotherapy [12]. Typically, it involves two to four intracavitary brachytherapy sessions following external beam radiotherapy [22]. Currently, 3D image–guided brachytherapy is the standard approach, necessitating treatment planning based on computed tomography or magnetic resonance imaging [13, 23]. The median duration of a single intracavitary brachytherapy session is reportedly 147 min, which is longer than that of 2D treatment [23]. Additionally, when intravenous anesthesia is used to alleviate patient anxiety and discomfort, additional medical staff and equipment are necessary, along with time for anesthesia induction and postprocedure monitoring [24]. Interstitial brachytherapy is considered when a bulky or irregularly shaped tumor complicates intracavitary brachytherapy [25]. This technique involves inserting applicators directly into the tumor and requires more advanced skills and medical resources than intracavitary brachytherapy [25]. Thus, radiotherapy for cervical cancer requires substantial medical resources, including a multidisciplinary team, trained staff, specialized equipment and sufficient treatment time. Advances in brachytherapy have increased these burdens.

These growing burdens highlight the importance of understanding the barriers to continued radiotherapy delivery. Several potential factors may exist, warranting further research. One concern is whether staffing levels for cervical cancer radiotherapy are sufficient relative to patient volume. Securing medical staff with brachytherapy expertise may be challenging, underscoring the need for training programs and multidisciplinary collaboration involving radiation oncologists, nurses, radiologic technologists and medical physicists. Establishing core hospitals that bring together medical staff and patients can help address staffing shortages, provide training opportunities and improve access through collaboration with nearby institutions. Ikushima *et al*. [13] reported that 47% of the brachytherapy facilities in Japan lack adequate training opportunities for residents, mainly because of fewer patients and instructor shortages.

Financial feasibility is another important consideration for continued brachytherapy delivery. If sustainability is in question, strategies such as centralizing treatment at core hospitals or securing financial support from prefectural authorities could be considered. The current reimbursement systems may not fully cover the costs associated with brachytherapy. A survey conducted in 2016 by Toita *et al*. [23] found that many physicians considered the current reimbursement for intracavitary brachytherapy to be insufficient. Similarly, Kinoshita *et al*. [26] found that four out of six brachytherapy facilities in Hokkaido Prefecture were considering discontinuing the service because of the financial burden of equipment renewal and maintenance.

Given that this study did not investigate obstacles to continued cervical cancer radiotherapy services, further data collection and evaluation are needed to clarify potential contributing factors. This study had certain limitations. The analysis was limited to institutions in Osaka. In 2020, Osaka had the second-highest number of patients with cervical cancer among all prefectures [4] with >10 facilities providing brachytherapy, while other prefectures had between 1 and 18 facilities, with a median of 2 [13]. Both the clinical need for and provision of radiotherapy for cervical cancer may vary by region. Further research using large-scale registry data is warranted to evaluate national trends and assess regional variation in the use of radiotherapy for cervical cancer treatment.

Hospital-based cancer registries recorded only initial treatment; therefore, the number of patients undergoing radiotherapy for recurrent tumors could not be estimated. These registries also lacked unique patient identifiers across institutions; thus, the proportion of patients receiving brachytherapy among those treated with radiotherapy could not be determined. In facility-based analyses, patients referred to other hospitals for brachytherapy were counted at both institutions, resulting in double counting. Since 2018, these registries have classified para-aortic lymph node metastases as regional lymph node involvement. This change may have contributed to the increased number of patients classified as having stage IIIC tumors. Accordingly, the observed increase in radiotherapy use among these patients may partly reflect current management practices for para-aortic lymph node metastases, in which radiotherapy may generally be preferred over surgery [6]. The study also did not account for temporary suspensions of patient acceptance related to equipment renewal or staff relocation. The COVID-19 pandemic may have influenced treatment selection and institutional case distribution. However, a 2021 survey by the Japan Society of Gynecologic Oncology [27], which investigated the effect of the COVID-19 pandemic, found that ~80% of institutions nationwide observed no change in the frequency of radiotherapy for gynecologic cancers.

In conclusion, this study revealed an increasing clinical need for cervical cancer radiotherapy in Osaka Prefecture, although the increase in patient numbers varied across facilities. This variation suggests that an uneven increase in patient numbers across institutions may compromise the long-term sustainability of cervical cancer radiotherapy services in the region. Further research could be valuable to evaluate the adequacy of staffing in facilities that provide brachytherapy, explore the financial feasibility of equipment modernization and maintenance, and assess the implications of brachytherapy centralization.

## DATA SHARING

The data were provided by the participating hospitals under a confidentiality agreement and are not publicly available.
